# Analysis of a Single Hemodialysis on Phosphate Removal of the Internal Fistula Patients by Mathematical and Statistical Methods

**DOI:** 10.1155/2013/856897

**Published:** 2013-12-24

**Authors:** Qiyao Yu, Yaling Bai, Junxia Zhang, Liwen Cui, Huiran Zhang, Jinsheng Xu, Chao Gao

**Affiliations:** ^1^Departments of Nephrology, The Fourth Hospital of Hebei Medical University, Shijiazhuang 050011, China; ^2^Departments of Radiation Oncology, The Fourth Hospital of Hebei Medical University, Shijiazhuang 050011, China

## Abstract

Chronic kidney disease related mineral and bone disease (CKD-MBD) is a worldwide challenge in hemodialysis patients. In china, the number of dialysis patients is growing but few data are available about their bone disorders. In the current study, we aimed to evaluate the effect of clinical factors on the serum phosphorus clearance in the 80 maintenance hemodialysis (MHD) patients. Six clinical factors were identified for their association with the serum phosphorus clearance using the analysis of Spearman's single linear correlation, including predialysis serum phosphate level, CRR, membrane surface area of the dialyzer, effective blood flow rate, the blood chamber volume, and hematocrit. In an overall multivariate analysis, pre-P, CRR, membrane SA, and Qb were identified as independent risk factors associated with the serum phosphorus clearance. In conclusion, HD could effectively clear serum phosphorus. The analysis of CRR might help to estimate serum phosphorus reduction ratio.

## 1. Introduction

In recent years, with the continuous improvement of the hemodialysis (HD) treatment and water quality, end-stage renal disease (ESRD) patients have led a gradually extended life, and their nutritional status has been improved. But at the same time, the calcium and phosphorus metabolism disorders and renal osteodystrophy are gradually becoming one of the long-term complications that affect the patient's quality of life and survival time, wherein hyperphosphatemia is a common complication of chronic kidney disease and is also one of the risk factors for death in dialysis patients [1]. Studies have shown that increased serum phosphorus and calcium-phosphorus product is one of independent risk factors for cardiovascular complications in patients with ESRD [2]. The risk of coronary artery calcification caused by each additional 1 mg/dL of phosphorus is equivalent to an additional 2.5 years for HD [3]. Mason and Shepler [4]**   **have reported that phosphorus levels of dialysis patients also have a direct relationship with mortality in patients undergoing maintenance HD. At present, few reports have been made about multivariate analysis of serum phosphorus clearance in single HD treatment. Therefore, we conducted this clinical observation, to further explore the factors related to serum phosphorus clearance.

## 2. Materials and Methods

### 2.1. Clinical Data

As study subjects, we enrolled those patients who were treated in blood purification center of the Fourth Hospital of Hebei Medical University between June 2010 and February 2012. Dialysis patients were considered eligible if they had been receiving HD for more than 3 months, showed no evidence of chronic or acute infections, inflammatory disorders, and malignancy, and were not using anti-inflammatory drugs for 3 months before enrollment. All of the 80 dialysis patients met K/DOQI diagnostic criteria for Stage 5 of chronic kidney disease (CKD) and received the treatment of hemodialysis. Among the dialysis cohort, 43 patients were diagnosed with primary chronic glomerulonephritis, 18 with diabetic nephropathy, 5 with polycystic kidney disease, 4 with IgA nephropathy, 3 with benign arteriolar nephrosclerosis, 3 with aristolochic acid nephropathy, 1 with lupus nephritis, 1 with obstructive kidney disease, 1 with gout kidney, and 1 with Alport syndrome. Patients were managed with German Fresenius 4008S dialysis machine and bicarbonate dialysate was used; dialysate flow rate was 500 mL/min, and effective blood flow rate was 200 to 300 mL/min. Five kinds of dialyzers were used for HD: polysulfone membrane dialyzer F6 (membrane surface area was 1.3 m^2^, and ultrafiltration coefficient was 5.5 mL/hr/mmHg) for 19 patients, double cellulose acetate membrane dialyzer CA-HP150 (membrane surface area was 1.5 m^2^, and ultrafiltration coefficient was 10.2 mL/hr/mmHg) for 36 patients, high-flux polysulfone membrane dialyzer F60 (membrane surface area was 1.3 m^2^, and ultrafiltration coefficient was mL/hr/mmHg) for 6 patients, high-flux polysulfone membrane dialyzer APS900 (membrane surface area was 1.8 m^2^, and ultrafiltration coefficient was 75 mL/hr/mmHg) for 9 patients, and cellulose triacetate membrane dialyzer CT-190 G (membrane surface area was 1.9 m^2^, and ultrafiltration coefficient was 17.4 mL/hr/mmHg) for 10 patients. There were two types of calcium concentration in dialysate: calcium concentration in dialysate was 1.25 mmol/L for dialysis in 16 patients and calcium concentration in dialysate was 1.5 mmol/L for dialysis in 64 patients. Before single HD treatment, phosphate binders (calcium) were not used and in the process of HD eating and urinating were avoided. The study was approved by the Human Tissue Research Committee of the Fourth Hospital of Hebei Medical University. All patients provided written informed consent for the collection of samples and subsequent analysis.

### 2.2. Preparation of Specimens

Before single HD blood samples were collected from the arterial end before connecting the arterial line and rinsing the puncture needle; specimens after HD were collected at the end of the dialysis; before collection the blood flow rate was reduced to 50 mL/min and specimens were collected from the arterial end closest to the patient, with 2 mL of blood collected each time. One hour after the collection blood serum calcium (tCa), serum phosphorus (P), serum creatinine (Cr), blood urea nitrogen (BUN), carbon dioxide combining power (CO_2_CP), and hematocrit (HCT) were tested in the clinical laboratory of our hospital. The patient's name, age, sex, type of dialyzer used in a single HD, membrane surface area, ultrafiltration coefficient, blood chamber volume, effective blood flow rate, and ultrafiltration volume were all recorded.

### 2.3. Main Equipments

We used some equipments such as Fresenius 4008S dialysis machine (Germany Fresenius Medical Care Co., Ltd.), polysulfone membrane dialyzer F60 (Germany Fresenius Medical Care Co., Ltd.), polysulfone membrane dialyzer F6 (Germany Fresenius Medical Care Co., Ltd.), double acetate fiber membrane dialyzer CA-HP150 (Germany Baxter Medical Goods Co., Ltd.), 3 diacetate fiber membrane dialyzer CAHP150 (Germany Baxtermedical Supplies Co., Ltd.), cellulose triacetate membrane dialyzer CT-190G (Germany Baxter medical Supplies Co., Ltd.) and polysulfone membrane 4 dialyzer APS900 (Japanese Asahi medical Co., Ltd.).

### 2.4. Calculation Formula

SRR(%) = [(CB − CA)/CB] × 100% CB was predialysis solute concentration (mmol/L) in the blood and CA was postdialysis solute concentration (mmol/L) in the blood. Reduction ratio (%) of the solute (serum phosphorus, creatinine and urea nitrogen) was calculated respectively.

### 2.5. Statistical Methods

SPSS 13 software package was used for data analysis, statistical description as well as statistical analysis was made in the statistical package, and charts were processed with Excel spreadsheet. Serum phosphorus before and after HD was expressed with mean ± standard deviation and paired *t*-test was used for comparison of their own before and after HD. *P* < 0.05 was considered statistically significant. 14 clinical parameters and serum phosphorus reduction ratio underwent linear regression analysis (dichotomous variables were assigned with values: gender: male = 0, female = 1; calcium concentration of the dialysate: 1.25 mmol/L = 0, 1.5 mmol/L = 1), respectively, and *P* < 0.05 was considered statistically significant; serum phosphorus reduction ratio was regarded as the dependent variable, correlated clinical parameters screened after univariate analysis were regarded as independent variables, multiple linear regression analysis (forward) was then made, and *P* < 0.05 was considered to be statistically significant.

## 3. Results

### 3.1. Clinical Characteristics of Hemodialysis Patients

A total of 80 hemodialysis patients were enrolled in this study including 52 males and 28 females, with age ranging from 25 to 85 and mean age of 56.1 years; they underwent maintenance HD for 3 to 80 months, with a mean time of 19.13 months. All patients were treated with arteriovenous fistula as vascular access for dialysis three times a week and 4.5 hours each time.

### 3.2. The Comparison of the Serum Phosphorus between Predialysis and Postdialysis in MHD Patients

Postdialysis serum phosphate level was lower than predialysis level (0.84 ± 0.21 mmol/L, 2.00 ± 0.53 mmol/L), which was statistically significant (*P* < 0.01) (see Table 1).

### 3.3. The Association of Clinical Characteristics with the Serum Phosphorus Reduction in MHD Patients

**Figure 1:**
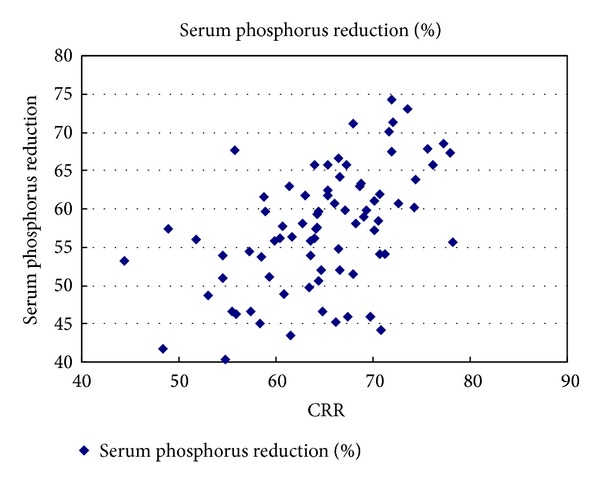
The correlation of predialysis serum phosphate and phosphate reduction ratio, *r* = 0.493, *P* < 0.05.

**Figure 2:**
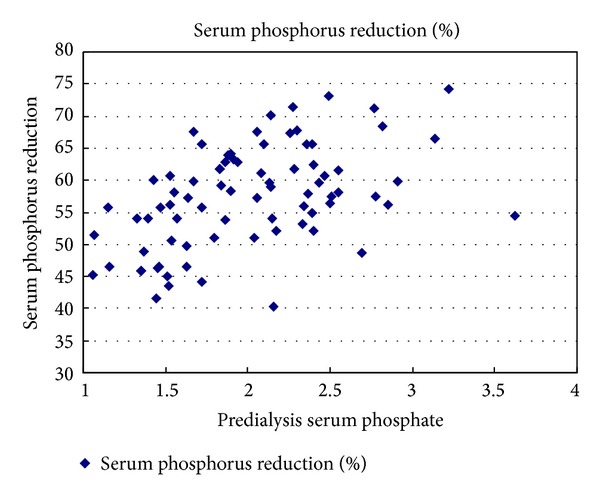
The correlation of creatinine reduction ratio and phosphate reduction ratio, *r* = 0.482, *P* < 0.05.

Serum phosphorus reduction ratio was positively correlated with predialysis serum phosphate, membrane surface area of the dialyzer, effective blood flow rate, CRR, and blood chamber volume but was negatively correlated with hematocrit (*r* = 0.493, 0.386, 0.368, 0.482, 0.303, and −0.225, resp., and *P* < 0.05) which can be seen in Figures 1 and 2 (see Table 2). The order of four independent factors related to serum phosphorus reduction was as follows based on their values (standardized coefficients beta): predialysis serum phosphate level, CRR, membrane surface area of the dialyzer, and effective blood flow rate (Std. Beta = 0.46, 0.439, 0.191, 0.177), and the above four factors were introduced into the equation to establish the multiple linear regression equation: *Y* = −12.156 + 6.821*X*_1_ + 0.493*X*_2_ + 7.307*X*_3_ + 0.051*X*_4_ (*R* = 0.759, *P* < 0.05; *Y* represents serum phosphorus reduction ratio, *X*_1_ represents predialysis serum phosphate, *X*_2_ represents CRR, *X*_3_ represents membrane surface area of the dialyzer, and *X*_4_ represents effective blood flow rate) (see Table 3).

## 4. Conclusion

### 4.1. The State of Serum Phosphorus in the MHD Patients

In normal human body phosphate is about 10 g/kg, while there is only 1% outside the cells. Phosphate in the bone accounts for 85%, including 14% intracellular phosphate and only 1% extracellular phosphate. The intracellular phosphate is mainly organophosphate. Phosphate in the plasma contains the organophosphate or form of phospholipids (70%) and about 30% of inorganic phosphate. Serum phosphorus that we measure usually refers to the inorganic phosphate, and normal plasma phosphorus ranges from 0.81 to 1.45 mmol/L (2.5~4.5 mg/dL). Phosphate is the small molecule toxin with molecular weight of less than 500 Da (Dalton). In the early stage of chronic renal failure, when glomerular filtration rate (GFR) is reduced to 40~80 mL/min/1.73 m^2^, phosphorus metabolism begins to change; when GFR is further reduced to 20~30 mL/min/1.73 m^2^, phosphorus begins to accumulate in the body, leading to persistent elevations of serum phosphorus, which results in hyperphosphatemia [5]. Although single hemodialysis significantly clears the serum phosphorus, the serum phosphorus ascends to the levels of prehemodialysis in a short time. It has been reported [6] that patients undergoing maintenance HD may develop hyperphosphatemia, which may be associated with increased protein intake, erythropoietin (EPO) therapy, intake of vitamin D, reduced physical activity, and so on. Hyperphosphatemia leads to further damage to residual renal unit in anatomy and function [7] and creates a vicious cycle, leading to secondary hyperparathyroidism [8, 9], renal osteodystrophy (ROD) [10], cardiovascular calcification [3], and hemodynamic abnormalities [11].

At  present, there are three main approaches  to the treatment of hyperphosphatemia. (1) *Controlling the intake of dietary phosphate*. Diet control can reduce the intake of phosphate, which is generally no more than 800 to 1000 mg per day [12]. However, it is difficult to control phosphorus of patients undergoing maintenance HD within the normal range by controlling diet clinically. (2) *Phosphate binders*. (a) Phosphate binders containing aluminum have the risk of aluminum poisoning, which can cause small-cell anemia, osteomalacia, dementia, and so on, so they are rarely used clinically. (b) Phosphate binders containing calcium, which will increase the absorption of calcium in the intestine, causing hypercalcemia and easily causing heart and vascular calcification when one suffers from hyperphosphatemia. (c) There have been new phosphate binders that are aluminum- and calcium-free at home and abroad, such as hydrochloric acid polyacrylamide (sevelamer, and trade name is Renagel) [13], but they are not widely used clinically in our country. (3) By blood purification not only nitrogenous waste can be cleared from the blood, such as urea nitrogen and creatinine, but also excess phosphorus in the body can be cleared. No matter what types of HD and dialyzer there are, serum phosphorus can be cleared significantly; different types of HD lead to different levels of serum phosphorus clearance. It has been reported that the hemodiafiltration (HDF) clear phosphorus content was more than that in conventional hemodialysis, so serum phosphorus clearance rate might be able to be improved by improving blood flow rate, increasing membrane surface area of the dialyzer, and improving the frequency of dialysis. Lindsay et al. [14] reported that daily short-term HD could increase serum phosphorus clearance and help prevent ROD and metastatic calcification [14]. In addition, long-term nocturnal HD could improve serum phosphorus clearance rate [15].

### 4.2. The Independent Risk Factors Associated with the Clearance of Serum Phosphorus in MHD Patients

HD is a process in which a solution exchanges solute with another solution through the semipermeable membrane, wherein small molecules such as urea nitrogen and phosphorus can be cleared mainly by applying the diffusion principle, while macromolecular toxins can be cleared mainly by applying the principle of convection and adsorption. The semipermeable membrane used in HD is called a dialysis membrane, the dialysis membrane is a major component of the dialyzer, and the physicochemical properties of the dialysis membrane can determine the effect of dialysis. There are three kinds of dialysis membrane commonly used clinically: regenerated cellulose membrane, modified cellulose membrane, and synthetic polymer membrane. It had been reported in China that polysulfone membrane and cellulose membrane showed no statistically significant difference in clearance of small molecule toxins, but in our purification center we chose polysulfone membrane, cellulose diacetate membrane and cellulose triacetate membrane, so in this study different membrane materials were not studied as clinical parameters.

Serum phosphorus is mainly cleared by diffusion as small molecule, and studies by many domestic and foreign scholars have confirmed that the general HD can effectively clear serum phosphorus. In this experiment, serum phosphorus reduction ratio was regarded as the observation index, to explore factors associated with serum phosphorus clearance in single HD treatment.The risk factors for serum phosphorus reduction included effective blood flow rate, membrane surface area, predialysis serum phosphorus level, vascular access, hematocrit, and dialysis frequency [16]. Clinical observation was made in our blood purification center about serum phosphorus reduction rate in 80 patients undergoing single HD treatment, and we got to know the order of independent correlative factors affecting serum phosphorus clearance as follows: predialysis serum phosphorus level, CRR, membrane surface area of the dialyzer, and effective blood flow rate (Std. Beta = 0.46, 0.439, 0.191, 0.177). That is, predialysis serum phosphorus level, membrane surface area of the dialyzer, and effective blood flow rate were independent factors affecting serum phosphorus clearance, while CRR was an independent correlative factor associated with serum phosphorus clearance.

Studies have shown that more than 80% of patients undergoing maintenance HD suffered from hyperphosphatemia. And in our blood purification center 50 out of 80 patients undergoing maintenance HD had hyperphosphatemia, accounting for 62.5% of the total number of cases. It is reported that predialysis serum phosphorus affected serum phosphorus clearance [17]. To analyze the reasons, we found it was mainly associated with the principle of dialysis for clearance. Diffusion was the irregular thermal motion of the molecules or particles of various substances; the solute was put in the solution, and after a certain period of time solutes in the entire solution would be uniformly distributed in solution. The driving force for diffusion was the concentration difference; in our purification center phosphate-free dialysate was used, and the larger concentration gradient of phosphorus was formed on both sides of the dialysis membrane, so that serum phosphorus was diffused to the side of dialysate. By Fick's law we knew that volumes of clearance by diffusion were proportional to solute concentration gradient; the higher predialysis serum phosphorus level was, the higher the concentration gradient between the blood side and the dialysate side would be. While in solute diffusion and transport, solute concentration gradient was the dynamic factor in the maintenance of diffusion. So, there would be the greater volume of solute clearance. It was also proved that controlling predialysis serum phosphorus level was particularly important.

### 4.3. The Effect of Membrane Surface Area of the Dialyzer on the Clearance of Serum Phosphorus in MHD Patients

Membrane surface area of the dialyzer is one of the parameters for the evaluation of the dialyzer; membrane surface area of the dialyzer r contact portions between the hollow fibers and the dialysate, and it is expressed as “m^2^”. Solute clearance was negatively correlated with thickness of dialysis membrane but was positively correlated with membrane surface area of the dialyzer. Studies on a new polysulfone membrane dialyzer have shown that blood flow rate, dialysate flow rate, and membrane surface area of the dialyzer are independent and significant factors that affect urea, creatinine, and serum phosphorus clearance. To increase the effective area of the dialysis membrane and the number of hollow fiber membranes could reduce the resistance of the retention layer of the blood side, increase exchange area between the blood and the dialysate, improve the diffusion rate, and have a significant effect on small molecule solute clearance and the clearance rate. The larger the membrane surface area, the faster the diffusion rate, the more small solutes get into the dialysate per unit time through the membrane hole, and the higher the clearance and the clearance rate will be. Accordingly, in the case of ensuring that the mechanical properties of the membrane could meet the dialysis conditions, the inner diameter of the membrane and wall thickness of the membrane should be reduced as much as possible, so as to increase the effective area of the dialysis membrane and to improve the clearance rate of the solute.

In HD, solute diffusion is influenced both by the membrane properties of the dialyzer and by immobile liquid layer between the blood side and the dialysate side. So-called immobile liquid layer refers to the fact that flow rates of the liquid are not the same in the process of flow in pipeline; the liquid in the middle flows faster, while the surrounding liquid flows slower. Near the wall there is a thin layer of liquid that hardly flows, which is referred to as the immobile liquid layer; the thicker the liquid layer, the lower the rate of solute diffusion. When the blood flow velocity is increased, the thickness of the immobile layer is decreased at the blood side, so that the diffusion rate of the small molecular substances is increased, helping reduce the transfer resistance of the blood side, and may improve dialysis efficiency and shorten the duration of dialysis without changing solutes transport coefficient (KOA) in the dialysis membrane; it also helps to improve the dialysis efficiency. However, with the increase in blood flow rate (>300 mL/min), the dialyzer cannot clear the solute with the same efficiency; that is, solute clearance rate cannot be increased indefinitely [18, 19] (e.g., blood flow rate was increased from 200 mL/min to 400 mL/min, and urea nitrogen clearance rate was increased by only 33%). Therefore, the impact of the pressure on the actual blood flow rate should be considered to guarantee the quality of dialysis. Serum creatinine is small molecule toxin with a molecular weight of 113 Da, and it is derived from endogenous creatinine (creatine decomposition of the muscles in the body) and exogenous creatinine (creatine in lean meat from animal food). The former is dominant and the latter is in a smaller proportion. Blood purification also can effectively clear serum creatinine; it has been found in the course of our study that CRR has a certain correlation with serum phosphorus reduction, which may be related to the unbalanced distribution of serum phosphorus and anhydride in the body such as blood, blood cells, and tissues.

In multivariate analysis we did not find high-flux hemodialysis, blood chamber volume, and hematocrit to be related to serum phosphorus reduction ratio. But high-flux hemodialysis is better than low-flux dialysis in the clearance of iPTH, and whether long-term observation has a certain effect on serum phosphorus clearance and prevention of hyperphosphatemia needs further prospective studies.

In short, hyperphosphatemia leads to complications such as ROD, cardiovascular calcification, and hemodynamic abnormalities, which all have a serious impact on the survival rate and quality of life of patients who undergo maintenance HD, so it is particularly important to control serum phosphorus levels. When it is tolerable for the patients, serum phosphorus clearance can be improved by increasing membrane surface area of the dialyzer and improving effective blood flow rate. At the same time high-flux dialyzers could be applied timely and hematocrit could be monitored regularly to understand serum phosphorus clearance. In clinical practice, we still lack a multicenter prospective controlled study with diverse samples, and various factors affecting serum phosphorus clearance are needed for further analysis and verification, so as to better guide clinical practice.

## Figures and Tables

**Table 1:** Comparison of serum phosphate between predialysis and postdialysis $\left(\overset{-}{x}\pm s\right)$.

Group	*n*	Serum phosphate
Predialysis	80	2.00 ± 0.53
Postdialysis	80	0.84 ± 0.21
*P*		<0.001

**Table 2:** Univariate analysis of clinical characteristics associated with the serum phosphorus from the dialysis patients.

Factors	Data	*r*	*P* value
Age (years)	56.10 ± 14.07	−0.003	0.976
Male	52	0.163	0.149
Female	28	0.163	0.149
Pre-P (mmol/L)	2.00 ± 0.53	0.493	<0.001
TCa (mmol/L)	2.16 ± 0.23	0.154	0.173
CO_2_CP (mmol/L)	18.73 ± 3.84	−0.033	0.771
HCT (%)	29.54 ± 6.05	−0.225	0.045
CRR (%)	64.80 ± 7.04	0.482	<0.001
Qb (mL/min)	247.00 ± 27.19	0.368	0.001
UF (mL)	2565.14 ± 1390.54	0.008	0.942
Dialysate calcium (1.25 mmol/L)	16	−0.143	0.204
Dialysate calcium (1.5 mmol/L)	64	−0.143	0.204
Membrane SA (m^2^)	1.52 ± 0.21	0.386	<0.001
TCV	88.73 ± 10.06	0.303	0.006
Kuf (mL/h·mmHg)	19.79 ± 29.80	0.164	0.146

UF: ultrafiltrate volume; Kuf: ultrafiltrate coefficient; TCV: blood chamber volume.

**Table 3:** The predictive variables for serum phosphorus clearance.

	Unstandardized coefficients *B*	Unstandardized coefficients Std. error	Standardized coefficients beta	*P*
Constant	−12.156	7.625		0.115
Pre-P	6.821	1.133	0.460	<0.001
CRR	0.493	0.086	0.439	<0.001
Membrane SA	7.307	3.126	0.191	0.022
Qb	0.051	0.024	0.177	0.034

*Note*. Pre-P: predialysis serum phosphate, CRR: creatinine reduction ratio, membrane SA: membrane surface area, and Qb: blood flow rate.
